# General anesthesia alters CNS and astrocyte expression of activity-dependent and activity-independent genes

**DOI:** 10.3389/fnetp.2023.1216366

**Published:** 2023-08-21

**Authors:** Zoeb Jiwaji, Nóra M. Márkus, Jamie McQueen, Katie Emelianova, Xin He, Owen Dando, Siddharthan Chandran, Giles E. Hardingham

**Affiliations:** ^1^ UK Dementia Research Institute, Edinburgh Medical School, The University of Edinburgh, Edinburgh, United Kingdom; ^2^ Centre for Discovery Brain Sciences, University of Edinburgh, Edinburgh, United Kingdom; ^3^ Centre for Clinical Brain Sciences, University of Edinburgh, Edinburgh, United Kingdom; ^4^ Department of Anaesthesia, Critical Care and Pain Medicine, Usher Institute, Edinburgh Royal Infirmary, Edinburgh, United Kingdom

**Keywords:** general anesthesia, transcriptional regulation, synaptic activity, astrocytes, neurodegenerating diseases

## Abstract

General anesthesia represents a common clinical intervention and yet can result in long-term adverse CNS effects particularly in the elderly or dementia patients. Suppression of cortical activity is a key feature of the anesthetic-induced unconscious state, with activity being a well-described regulator of pathways important for brain health. However, the extent to which the effects of anesthesia go beyond simple suppression of neuronal activity is incompletely understood. We found that general anesthesia lowered cortical expression of genes induced by physiological activity *in vivo*, and recapitulated additional patterns of gene regulation induced by total blockade of firing activity *in vitro*, including repression of neuroprotective genes and induction of pro-apoptotic genes. However, the influence of anesthesia extended beyond that which could be accounted for by activity modulation, including the induction of non activity-regulated genes associated with inflammation and cell death. We next focused on astrocytes, important integrators of both neuronal activity and inflammatory signaling. General anesthesia triggered gene expression changes consistent with astrocytes being in a low-activity environment, but additionally caused induction of a reactive profile, with transcriptional changes enriched in those triggered by stroke, neuroinflammation, and Aß/tau pathology. Thus, while the effects of general anesthesia on cortical gene expression are consistent with the strong repression of brain activity, further deleterious effects are apparent including a reactive astrocyte profile.

## Introduction

General anesthesia (GA) is a common clinical intervention, with nearly 230 million surgical procedures taking place under GA across the world every year (Weiser et al., 2008). Exposure to anesthetic agents has been implicated in increasing neuronal apoptosis (Yon et al., 2005), worsening dementia neuropathology (Xie et al., 2008), increasing perioperative delirium (Robinson and Eiseman, 2008) and driving short and long-term memory impairment in humans and animals (Dutton et al., 2002; Culley et al., 2004; Pan et al., 2011; Bratzke et al., 2018). However, the molecular pathways by which anesthesia induces these deleterious effects is unclear.

Although the exact mechanisms by which GA agents work are yet to be fully elucidated, they are widely considered to suppress neuronal firing (Ries and Puil, 1993) via GABA receptor agonism or NMDA receptor antagonism (Chung et al., 2021). Anesthesia induces burst suppression on EEG (Lukatch et al., 2005), with both acute and long-lasting exposure to anesthesia being shown to dysregulate expression of selected activity-regulated immediate early genes (Upton et al., 2020). GA agents also have wider consequences on the CNS beyond those only through modulation of neuronal firing. They bind alternative non-GABA or non-NMDA receptors (Son, 2010); have global membrane effects via action on lipid-rafts (Pavel et al., 2020) and alter CNS blood flow (Slupe and Kirsch, 2018). Previous work by ourselves and others has established that neuronal activity is a key regulator of CNS transcription, driving cellular and circuit-level changes necessary for memory and neuronal health (Bell and Hardingham, 2011; Deighton et al., 2014; Baxter et al., 2015; Hasel et al., 2017). However the extent to which anesthesia alters activity-regulated pathways important for brain health, as well as consequences on non-activity dependent pathways, was undetermined.

Beyond altering neuronal transcription, neuronal activity also has consequences on gene expression in nearby non-neuronal cells. Previously, we found that neuronal activity altered transcription in astrocytes (Hasel et al., 2017), the most common non-neuronal cell in the brain. These cells play important roles for CNS function including glutamate clearance (Anderson and Swanson, 2000), ionic regulation (Hertz and Chen, 2016), metabolic (Weber and Barros, 2015; Hasel et al., 2017) and antioxidant homeostasis (Jiwaji and Hardingham, 2022). We determined that neuronal activity upregulated astrocyte glycolytic gene expression, hence increasing the capacity of astrocytes to provide metabolic support to neurons (Hasel et al., 2017). However, the extent to which neuronal activity regulates astrocyte transcription *in vivo*, and whether anesthesia, by suppressing neuronal firing, altered previously identified astrocyte activity-dependent homeostatic pathways was unknown. Furthermore, astrocytes express GABA receptors (Chung et al., 2021) and anesthesia directly alters astrocyte calcium signaling (Thrane et al., 2012), lactate production (Hadjihambi et al., 2020), and gap junction formation (Liu et al., 2016). However, the extent to which anesthesia (via activity-dependent or activity-independent pathways) alters astrocyte transcriptional pathways was unknown.

In this study, we aimed to determine how anesthesia alters CNS and astrocyte transcriptional pathways. As anesthesia is associated with deleterious consequences, we aimed to identify consequences on CNS and astrocyte transcriptional pathways important for brain health. In addition, we aimed to identify the extent to which anesthesia-mediated gene expression changes overlapped with effects mediated through neuronal activity suppression vs. activity-independent effects in a whole brain situation.

## Materials and methods

### Animal husbandry

All procedures described were performed in compliance with the UK Animals (Scientific Procedures) Act 1986 and University of Edinburgh regulations, and carried out under project license numbers 70/9008 and P1351480E. Mice were group-housed in environmentally-enriched cages within humidity and temperature controlled rooms, with a 12-h light dark cycle (unless otherwise stated), with free access to food and water.

### Transgenic mouse lines

The Aldh1l1-EGFP-Rpl10a transgenic mouse line expressing an EGFP-tagged ribosomal protein l10a under the astrocyte-specific Aldh1l1 promoter was generated as previously described (Doyle et al., 2008) and re-derived on a C57B/6 background from frozen sperm imported from Jackson Laboratories (Mouse Strain Number—030248). Animals were utilised as heterozygotes in all experiments and mixed-sex unless otherwise stated.

### Prolonged anesthesia

Six week old mice were induced in an anesthesia chamber with 4% isoflurane in oxygen, followed by maintenance anesthesia via facemask with 1.5% isoflurane. Mice were actively warmed to 36°C, and temperature, pulse, respiratory rate and oxygen saturations continuously monitored and maintained within normal physiological range using the Physiosuite monitoring system (Kent Scientific). 0.1 mL subcutaneous warmed sterile 0.9% saline solution was administered every 1–2 hrs to maintain hydration. Following anesthesia, mice were culled and whole neocortices rapidly dissected for RNAseq or TRAP analyses.

### Visual deprivation and light stimulus

Four week old mice were transferred from standard lighting conditions to a temperature and ventilation controlled light-proof cabinet and housed in constant darkness for 6 days. All checks during this period were carried out in complete darkness using night-vision equipment. Mice were then exposed to a further 24 h of darkness or 24 h of continuous light, following which they were decapitated and visual cortices rapidly dissected.

### Primary cortical neuronal culture

Primary cortical neurons were cultured from E17.5 CD1 mice embryos using a modified version of the protocol described by Bading and Greenberg, 1991. Cortices were dissected following decapitation in dissociation medium (DM) (mM: 81.8 Na2SO4, 30 K2SO4, 5.84 MgCL2, 0.252 CaCl2, 1 HEPES, 0.001% Phenol Red, 20 Glucose, 1 kyurenic acid, adjusted to pH 7.35 with NaOH). Tissue was enzymically digested by incubation with DM with 10 units/mL papain enzyme (Worthington Biochemicals) for 40 min with regular mixing, followed by two washes with DM and two washes with Neurobasal-A medium (Thermofisher) supplemented with 1% rat serum (Envigo), 1 x B-27 supplement (Thermofisher), 1 x Antimicrobial-Antifungal supplement (premixed penicillin, streptomycin and amphotericin B- Thermofisher) and 1 mM glutamine (Sigma). Following washing, cortices were mechanically dissociated with repeated mixing using a 5 mL serological pipette and the resulting cell-suspension diluted with Opti-MEM (Life Technologies) supplemented with glucose (20 mM) and Antimicrobial-Antifungal to achieve a final tissue concentration of 0.14 mouse cortical hemispheres.

0.5 mL of cell suspension was added to 24-well tissue-culture plates pre-coated with 0.5 mL of water with 0.5% laminin from mouse Engelbreth-Holm-Swarm sarcoma cells (Roche) and 1.33% poly-D-lysine (Sigma). Plates were incubated for 2.5 h at 37°C in a humidified 5% CO2 incubator. After this, plate medium was aspirated (along with dead or non-adherent cells) and replaced 1 mL Neurobasal-A medium supplemented with 1% rat serum, B-27, Antimicrobial-Antifungal and glutamine and 4.3 μM of the antimitotic cytosine B-D-arabinofuranoside hydrochloride (AraC) to prevent glial growth. Cultures were subsequently fed at day *in vitro* (DIV) 4 with 1 mL of Neurobasal-A medium supplemented with 1% rat serum, B-27, Anti-Anti, glutamine and AraC.

Suppression of synaptic activity was achieved by changing cultured cells into serum-free base-medium (10% minimum essential media (MEM, Life Technologies) and 90% salt–glucose–glycine (SGG) containing in mM: 114 NaCl, NaHCO3, 5.292 KCl, 1 MgCl2, 2 CaCl2, 10 HEPES, 1 Glycine, 30 Glucose, 0.5 sodium pyruvate, 0.1% Phenol Red) with 100 nM tetradotoxin (TTX, Tocris) on DIV9 for 24 h to inhibit neuronal action potential firing. Control conditions were placed into serum-free base-medium only.

### RNA extraction

RNA extraction was carried out using the High Pure RNA Isolation Kit (Roche) as per manufacturer’s instructions. Three wells of a 24 well cell-culture plate were lysed per sample and transferred into glass-fibre filter column assemblies. Following DNAse incubation for 15 min, the columns were washed, dried and RNA eluted in 30 μL of elution buffer.

Low-yield RNA extraction (for example, following ribosomal pulldown) was carried out using the Absolutely RNA Nanoprep kit (Agilent) as per manufacturer’s instructions. Samples were lysed in 100 μL of lysis buffer with β-mercaptoethanol; mixed with equal volume of 80% sulfolane and added to silica-based fibre filter columns. DNAse treatment was carried out for 15 min; columns were washed and dried, and RNA eluted in 20 μL of pre-warmed 60°C RNAse free water.

### Translating ribosome affinity purification (TRAP)

Isolation of cell-type specific translating mRNA was carried out as previously described (Heiman et al., 2014). Briefly: tissue or cells were lysed in ice-cold lysis buffer (in mM: 20 HEPES, 10 MgCl_2_, 150 KCl, 0.5 DTT) along with 100 μg/mL cyclohexamide (Sigma), cOmplete ULTRA protease inhibitors (Sigma) and RNAse inhibitors (Superasin—Life Technologies; RNAsin—Promega). Cell lysate was centrifuged to clear debris and solubilised with 1% NP40 and 30 mM 1,2-diheptanoyl-sn-glycero-3-phosphocholine (DHPC—Avanti Polar Lipids). Following further centrifugation, supernatants were added to pre-prepared anti-GFP (19C8 and 19F7 antibodies—Sloan Kettering Memorial Centre) coated magnetic beads (Dynabeads MyOne Streptavadin T1—Life Technologies). Following immunoprecipitation overnight at 4°C with continuous rotation, beads were washed four times. Cell-type specific mRNA, attached to immunoprecipitated GFP-tagged ribosomes, was isolated and purified using the Agilent Nanoprep kit described above. 50 μL of solubilised lysate (“input sample”—representing total RNA) was taken prior to immune-precipation and spun overnight at 4°C (same conditions as TRAP samples) before RNA-purification with TRAP samples.

### Reverse-transcription PCR (rtPCR)

cDNA was generated using the Transcriptor First Strand cDNA Synthesis Kit (Roche). 7 μL of RNA was added to the RT and buffer mixture prepared with random hexamers and oligoDT primers as per kit instructions, and rtPCR carried out with the following programme: 10 min at 25°C, 30 min at 55°C and 5 min at 85°C.

### Quantitative PCR (qPCR)

qPCRs were performed on a Mx3000P QPCR machine (Agilent Technologies) using the FastStart Universal SYBR Green QPCR Master (Rox) (Roche) reagent. 6 ng of cDNA was used for each reaction and all qPCR results were carried out in duplicate or triplicate, along with no template controls and no RT controls where appropriate. The following cycling programme was used: 10 min at 95°C; 40 cycles of: 30 s at 95°C, 40 s at 60°C (with fluorescence detection), 1 min at 72°C; ending with dissociation curve: 1 min at 95°C and 30 s at 55°C with a ramp up to 30 s at 95°C with fluorescence detection. All data was normalised to house-keeping gene controls (*Rpl13a*). Table 1.

**TABLE 1:** Primer sequences.

Gene	Forward (5′–3′)	Reverse (5′–3′)
*Aif1*	GCA​ATG​ATG​AGG​ATC​TGC​C	CCA​CTG​GAC​ACC​TCT​CTA​ATT​AAT​C
*Aldh1l1*	GTTGCTAGCCCAGAGCC	GGA​ACT​TAA​ACA​CGG​GCA​C
*Arc*	AGT​GGT​GGG​AGT​TCA​AGC​AG	TCC​TCA​GCG​TCC​ACA​TAC​AG
*Bdnf*	AAA​GTC​CCG​GTA​TCC​AAA​GG	CTT​ATG​AAT​CGC​CAG​CCA​AT
*Cx3cr1*	CTGGTGGTCTTTGCCTTC	GCA​CTT​CCT​ATA​CAG​GTG​TCC
*Eno2*	GCC​ATC​TCC​TGT​AAC​TCT​CC	ATT​CTG​TAA​AGT​TCC​GAG​CTT​C
*Gfap*	GCA​AAA​GCA​CCA​AAG​AAG​GGG​A	ACA​TGG​TTC​AGT​CCC​TTA​GAG​G
*Mbp*	CCA​GTC​TAA​TAA​TGT​CCA​TCG​AC	CAG​ATT​AAC​AAG​ATG​CAG​TAT​TGG
*Mog*	AAA​GAA​TAC​CGA​CCA​GAG​AAA​TAC	CAC​ATT​GGT​TCT​CAG​AGA​AAT​AAG
*Npy*	AGA​CCT​CTT​AAT​GAA​GGA​AAG​CAC	CAG​GCA​GAC​TGG​TTT​CAG​G
*Rpl13a*	GAT​GAA​TAC​CAA​CCC​CTC​C	CGA​ACA​ACC​TTG​AGA​GCA​G

### RNA-sequencing

Total RNA sequencing was performed by Edinburgh Genomics using Trueseq Stranded Total RNA V2 library preparation along with next-generation sequencing on the Illumina Novaseq 6000 platform. At-least 1 μg RNA per sample was utilised, with RNA-integrity number (RIN) > 7. TRAP-sequencing (along with sequencing of matched inputs where appropriate) was performed by Cambridge Genomic Services, utilising the Clonetech - SMART-Seq v4 Ultra Low input RNA library preparation, along with sequencing on the Illumina Nextseq 500 platform. At least 1 ng RNA was used per sample with RIN > 7.

### Bioinformatics

RNA-seq reads were mapped using the STAR RNA-seq read aligner, version 2.5.3a, using default parameters. Tables of per-gene read counts were generated from the mapped read BAM files with featureCounts, version 1.5.2, using the -p parameter to specify fragments will be counted instead of reads in cases where the input data is paired-end, and the -s parameter set accordingly as the input data is unstranded or reversely-stranded. Gene annotations in GTF format were downloaded from Ensembl version 90. Differential gene expression analysis was performed in R using DESeq2, version 1.18.1, with betaPrior set to TRUE to perform fold change shrinkage, and the significance threshold calculated at a Benjamini–Hochberg adjusted *p*-value < 0.05, unless otherwise stated within the text. Gene ontology enrichment analysis was performed using the R package topGO, version 2.30.1, with the default “weight01” algorithm for dealing with the GO graph structure, and using Fisher’s exact test statistic. Basic statistics about the RNA-sequencing data are provided in Supplementary Data S1.

## Results

1. General anesthesia alters a large programme of cortical transcription and suppresses known activity-regulated immediate early genes.

**FIGURE 1:**
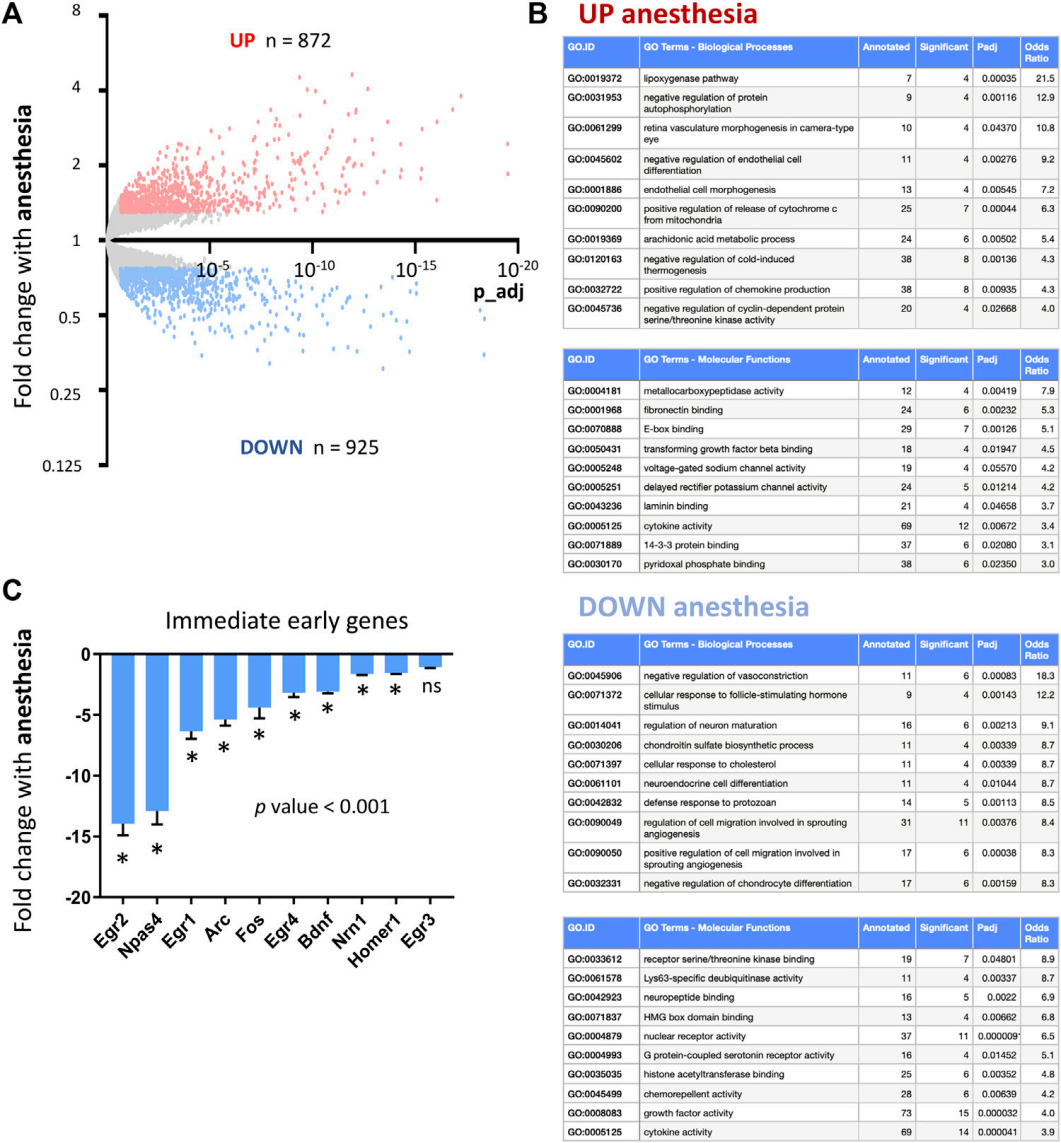
Consequences of anesthesia on cortical transcription. **(A)** RNAseq used to determine cortical gene expression changes in mice exposed to 6 h isoflurane anesthesia. Genes significantly induced (red) and repressed (blue) are highlighted (expression cut-off > 1 FPKM, > 1.3 FC up or down, *p* values adjusted for multiple testing by the Benjamini–Hochberg procedure to give a false discovery rate of 5% (p_adj < 0.05) here and in all RNA-seq analyses; *n* = 5 animals per condition). **(B)** Ontological analysis of genes induced and repressed by anesthesia. Top ten most significantly enriched pathways are shown, with pathways with less than four significant genes or not relevant to tissue type omitted. **(C)** Fold change expression of immediate early and activity-regulated neuroprotective genes in mice undergoing 6 h isoflurane anesthesia v controls (Mean ± SEM fold change anesthesia v control, N = 5 animals per condition, *p_adj < 0.05, overall *p*-value < 0.001, 1-way ANOVA, all gene expression data shown here and elsewhere, unless otherwise stated, determined using RNA-sequencing).

We first set out to define how general anesthesia altered cortical transcription. Mice were exposed to 6 h isoflurane anesthesia, following which neocortices were dissected for RNA extraction and gene expression analysis using RNA-sequencing (RNAseq). Differential gene expression analysis revealed that anesthesia altered the expression of hundreds of CNS genes (Figure 1A; Supplementary Data S2-1). Ontology analysis of upregulated and downregulated genes (Figure 1B) highlighted anesthesia-associated upregulation of deleterious pathways including those for mitochondrial stress responses associated with apoptosis through release of cytochrome C (*Hrk, Bid, Bbc3, Pmaip1, Plaur, Fas, Bm*f) and inflammation-induced responses including lipoxygenase synthesis (*Alox12b, Aloxe3, Alox8, Pla2g3*) and chemokine production (*Il4ra, Oas1c, Ticam1, Cyr61, Havcr2, Tlr7, Alox8, Trpv4*). Important pathways downregulated included those involved in the control of vasculature (*Apln, Arhgap42, Cx3cl1, Ahr, Dock5, Dusp5*) and neural maturation (*Bcl2, Dleu2, Lrrk2, Mir212, Mir132, Ngf*) and differentiation (*Bdnf, Notch1, Hdac5, Tmem119*)*.*

The mechanism of action for most anesthesia agents is considered to be via inhibition of neuronal firing by GABA activation and NMDA receptor inhibition. We therefore investigated the consequences of anesthesia on immediate early genes previously described to be upregulated by neuronal firing (Lin et al., 2008; Lacar et al., 2016). We found that anesthesia is a potent suppressor of immediate early genes including down-regulating activity-regulated genes that have previously been described to have neuroprotective or pro-synaptogenesis properties (*Bdnf*, *Arc*, *Npas4*, *Homer*) (Lin et al., 2008; Kim et al., 2018; Pollina et al., 2023) (Figure 1C, qPCR validation of selected IEGs in Supplementary Figure S11).

2. Visual sensory-deprivation and light re-exposure stimulation to determine CNS activity-regulated genes.

Neuronal firing is a key regulator of CNS transcription (Papadia et al., 2008; Léveillé et al., 2010; Bell and Hardingham, 2011; Baxter et al., 2015; Qiu et al., 2020). Having identified that anesthesia suppresses a limited set of markers previously described to be associated with neuronal firing, we next set out to determine more completely the extent to which anesthesia alters the expression of genes that are controlled by neuronal activity.

**FIGURE 2:**
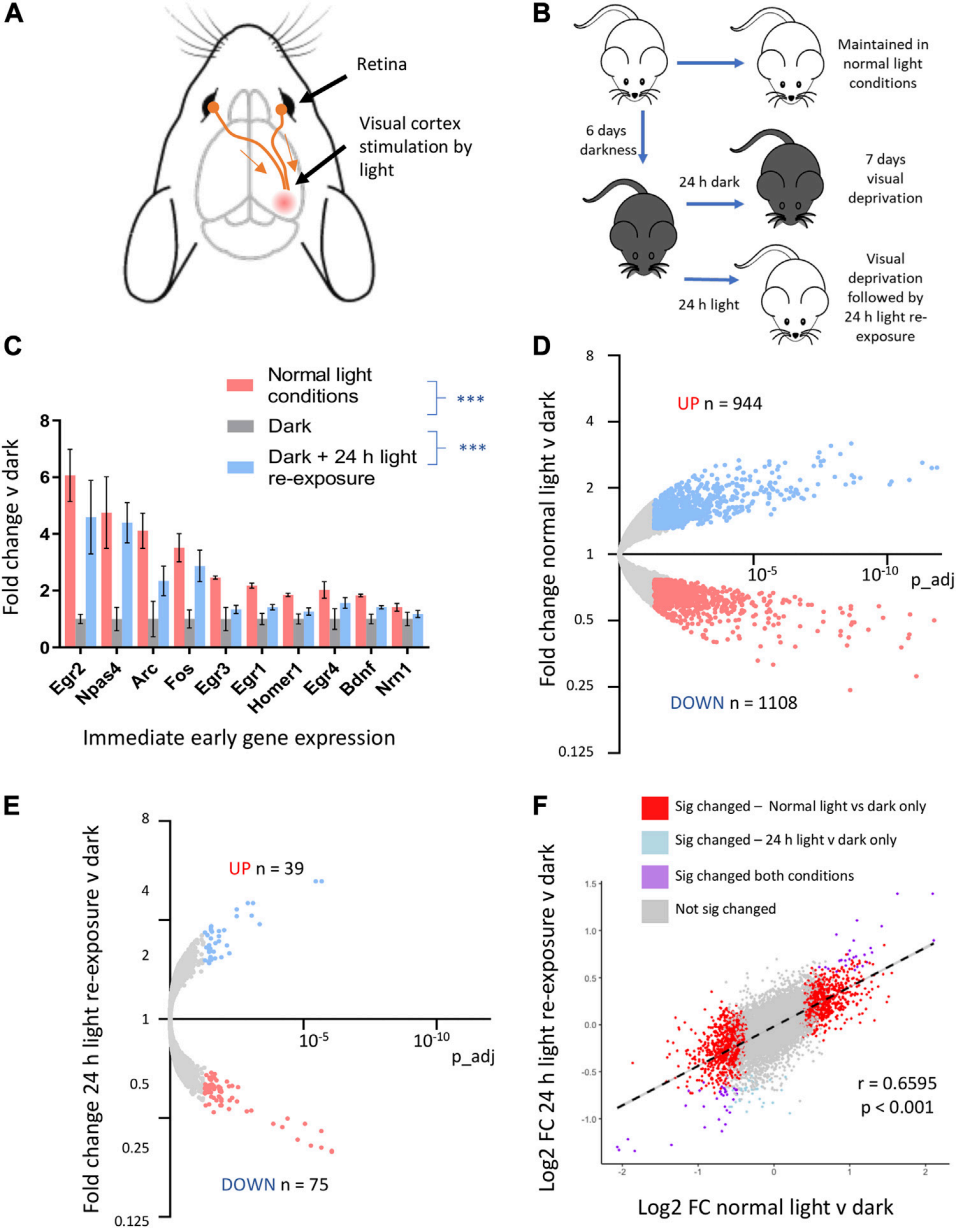
Visual sensory deprivation and light-re-exposure determines activity-mediated CNS gene expression. **(A)** Visual cortex innervation from the retina allows manipulation of neuronal activity by exposure to conditions of light v darkness. **(B)** Schematic of experimental protocol. Mice were kept in normal light conditions, total darkness for 7 days, or total darkness for 6 days followed by 24 h of light-re-exposure. **(C)** Fold change immediate early gene expression in mice exposed to normal light conditions, 7 days of darkness or 6 days of darkness followed by 24 h light re-exposure v mean expression in mice kept in darkness (mean ± SEM, ***p-value < 0.001, 1-way ANOVA, N = 4 mice per condition here and in all experiments involving visual sensory deprivation and light-stimulation). **(D)** RNAseq analysis reveals the consequences of visual sensory deprivation on visual cortex gene expression. Genes significantly induced by normal light conditions v darkness (red) and repressed (blue) are highlighted (expression cut-off > 1 FPKM, FC up or down > 1.3, p_adj < 0.05). **(E)** 24 h light re-exposure following sustained darkness alters CNS gene expression. Genes significantly induced or repressed by 24 h light re-exposure v darkness (red) and repressed (blue) are highlighted (expression cut-off > 1 FPKM, FC up or down > 1.3, p_adj < 0.05). **(F)** Gene expression changes after 24 h light re-exposure v darkness in mice correlated with changes identified in mice kept in normal light conditions v darkness. Significantly altered genes in either paradigm are plotted to show log2 fold change expression in normal light v darkness and 24 h light-exposure v darkness (R2 and *p*-value from Pearson correlation).

To better define CNS genes regulated *in vivo* by neuronal activity, we used a visual sensory deprivation and light-exposure paradigm. This allowed us to create conditions of altered neuronal activity in the visual cortex (Figure 2A). Mice were kept in complete darkness for 7 days to suppress visual cortex neuronal activity, and gene expression changes were compared to mice kept in normal light conditions (standard 12:12 light: dark cycle), as well as those exposed to complete darkness for 6 days and then re-exposed to light for 24 h (light re-exposure) (Figure 2B).

We confirmed that the expression of known immediate-early genes is reduced in animals kept in darkness for 7 days and elevated in animals kept in darkness and then re-exposed to light for 24 h (Figure 2C, qtPCR validation of selected IEGs in Supplementary Figure S11). Using RNAseq followed by differential gene expression-analysis, we identified visual cortex genes up and downregulated by normal light v darkness (Figure 2D; Supplementary Data S2-2). Light re-exposure following darkness significantly altered a smaller number of genes as compared to mice always kept in normal light conditions (Figure 2E; Supplementary Data S2-3). However, there was a strong correlation in the directionality and magnitude of change between those genes significantly changed in the context of normal light-conditions v darkness with those changed by light re-exposure (Figure 2F).

3. Anesthesia suppresses visual stimulation-mediated gene expression.

**FIGURE 3:**
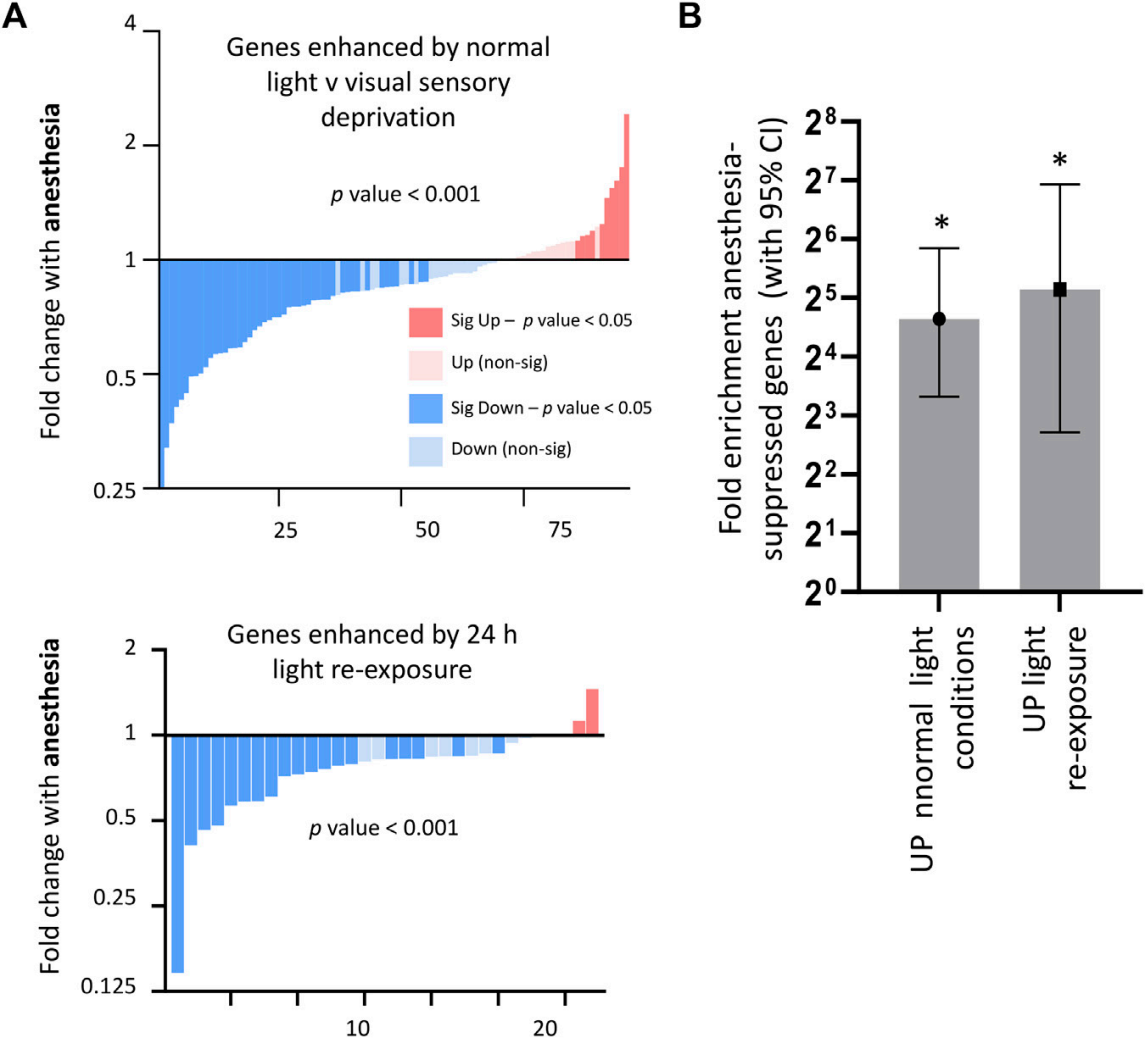
Consequences of anesthesia on genes regulated by visual sensory stimulation. **(A)** Anesthesia overall suppresses genes enhanced in normal light conditions v darkness (expression cut-off > 1 FPKM, p_adj < 0.05, FC > 2), or enhanced by light-re-exposure v darkness (expression cut-off > 1 FPKM, p_adj < 0.05, FC > 1.5). Fold change 6 h anesthesia v control (expression cut-off > 1 FPKM, *p*-value < 0.001, ratio paired 2-tailed *t*-test). **(B)** Visual activity-regulated genes are significantly enriched for genes suppressed by anesthesia. Genes induced > 2 fold by normal light conditions or > 1.5 fold in light re-exposure (expression cut-off > 1 FPKM, p_adj < 0.05) taken and enrichment analysis performed using genes found to be downregulated (expression cut-off > 1 FPKM, p_adj < 0.05, > 2 FC) by anesthesia. Fold enrichment is shown, and 95% confidence interval (CI) of the fold enrichment depicted by the error bar. (*p < 0.05 two-sided Fisher’s exact test).

Given that anesthesia is proposed to act as an inhibitor of neuronal firing, we next determined the extent to which genes altered by visual sensory experience were also regulated by anesthesia. We found, as predicted, that anesthesia overall significantly suppressed genes upregulated by visual sensory experience. This was observed both in conditions of normal light v darkness and light re-exposure v darkness (Figure 3A), with light-stimulated gene-sets being significantly enriched for genes found to be suppressed by anesthesia (Figure 3B). However, those genes suppressed by light-stimulation were not overall upregulated by anesthesia as predicted (Supplementary Figure S3).

4. Total neuronal activity blockade *in vitro* reveals an extended set of activity-regulated genes altered by anesthesia beyond those demonstrated by visual-sensory deprivation.

We next set out to better understand the identified discordance between how anesthesia altered those genes enhanced or suppressed by visual sensory stimulation. One possible reason was that the level of activity suppression achievable in the visual cortex by darkness was limited. Indeed, the mouse visual cortex receives innervation from other cortical areas, and therefore even in darkness there exists an element of background neuronal firing (Keller et al., 2012).

Therefore, we asked to what extent anesthesia altered CNS gene-expression by suppressing neuronal activity to a level beyond that possible by visual sensory deprivation alone. To determine genes altered by complete neuronal blockade, we used an *in vitro* primary mouse cortical neuronal culture system as previously described (Baxter et al., 2021). Primary mouse cortical neurons were cultured until day *in vitro* (DIV) 10 (by which time they have developed mature synapses with robust synaptic activity (Hasel et al., 2017)), and then placed into fresh medium containing 100 nM tetrodotoxin (TTX)—a voltage-gated sodium channel blocker that completely stops neuronal firing. Following neuronal activity blockade for 24 h, cells were lysed and RNA extracted for gene expression analysis via RNAseq, and compared to control neurons kept in TTX free medium.

**FIGURE 4:**
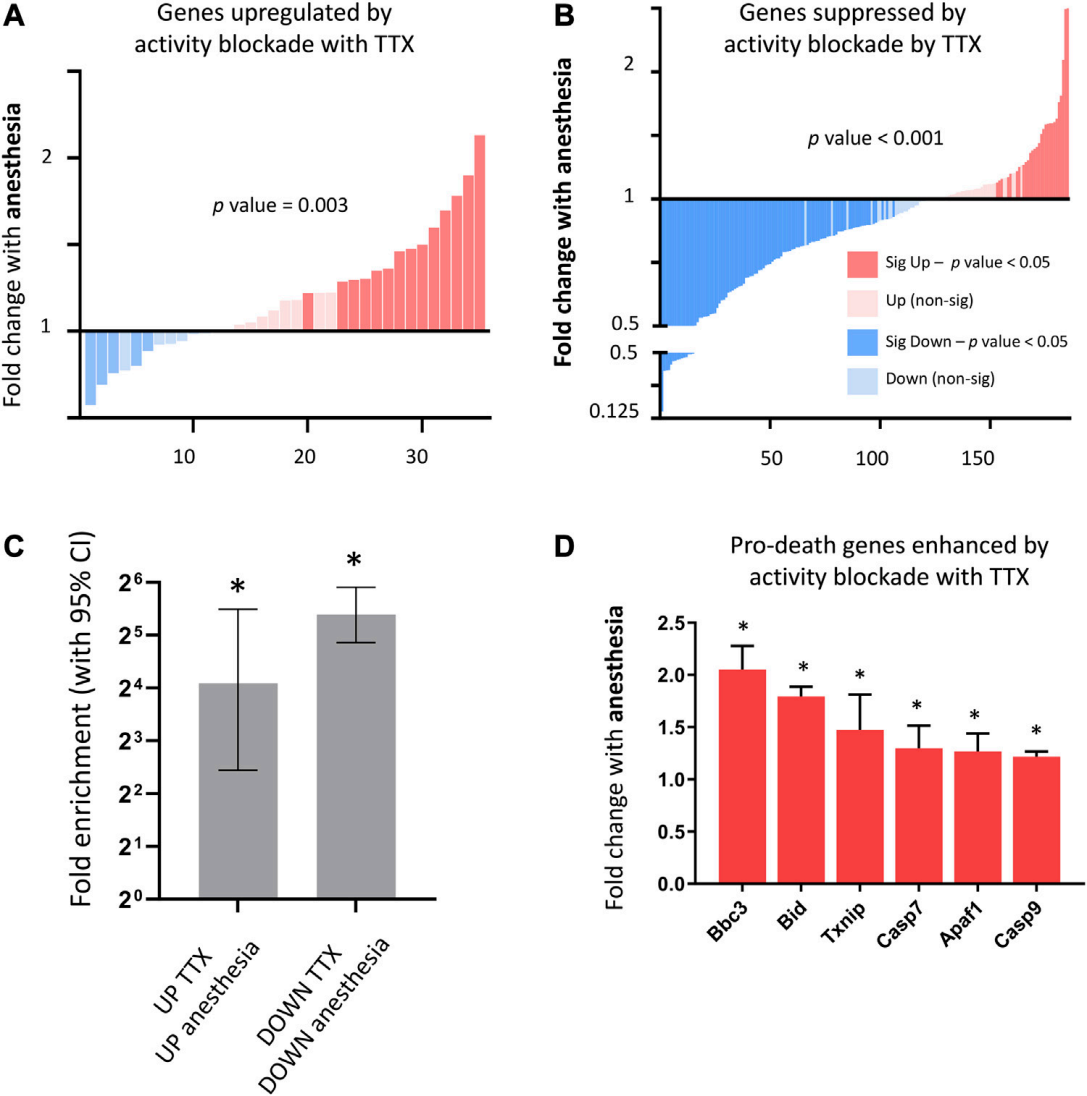
Consequences of anesthesia on genes altered by neuronal-activity blockade *in vitro*. **(A)** Anesthesia overall upregulates genes enhanced by neuronal activity blockade (genes upregulated following 24 h 100 nM TTX in neuronal culture, expression cut-off > 1 FPKM, FC > 2, p_adj < 0.05). Fold change anesthesia v control shown (expression cut-off > 1 FPKM, ratio paired 2-tailed *t*-test). **(B)** Anesthesia overall downregulates genes enhanced by neuronal activity blockade. Fold change anesthesia v control shown (expression cut-off > 1 FPKM, ratio paired 2-tailed *t*-test). **(C)** Genes altered up or down by neuronal activity blockade *in vitro* are significantly enriched for genes respectively enhanced or suppressed by anesthesia. Enrichment analysis performed on genes induced or suppressed by TTX using genes found to be upregulated and downregulated (expression cut-off > 1 FPKM, p_adj < 0.05, > 1.5 FC) by anesthesia. Fold enrichment is shown, and 95% confidence interval (CI) of the fold enrichment depicted by the error bar. (*p-value < 0.05 two-sided Fisher’s exact test). **(D)** Activity-regulated pro-death genes found to be upregulated by neuronal activity blockade *in vitro*, are also upregulated by anesthesia. Expression of pro-death genes upregulated by *in vitro* neuronal activity blockade using TTX (expression cut-off 1 FPKM, p_adj < 0.05) following 6 h isoflurane anesthesia (Mean ± SEM fold change expression anesthesia v control, N = 5 animals per condition, *p_adj < 0.05, overall *p*-value < 0.001, 1-way ANOVA).

Using differential gene expression, we identified genes altered by complete neuronal activity blockade (Supplementary Data S2-4) and determined that overall anesthesia strongly upregulated genes enhanced by complete neuronal activity blockade (Figure 4A), and strongly downregulated genes suppressed by neuronal activity blockade (Figure 4B), finding that genes increased or decreased *in vitro* by neuronal activity blockade were significantly enriched for genes upregulated or downregulated respectively by anesthesia (Figure 4C). Genes upregulated by TTX blockade included previously identified activity-suppressed pro-death genes (Léveillé et al., 2010). These genes were also significantly upregulated by anesthesia (Figure 4D), but not in the context of visual sensory deprivation (data not shown).

Overall, these data suggest that anesthesia drives suppression of neuronal firing beyond that achieved by visual sensory deprivation, leading to upregulation of detrimental genes normally suppressed by neuronal activity.

5. Anesthesia alters the expression of genes not regulated by neuronal activity.

**FIGURE 5:**
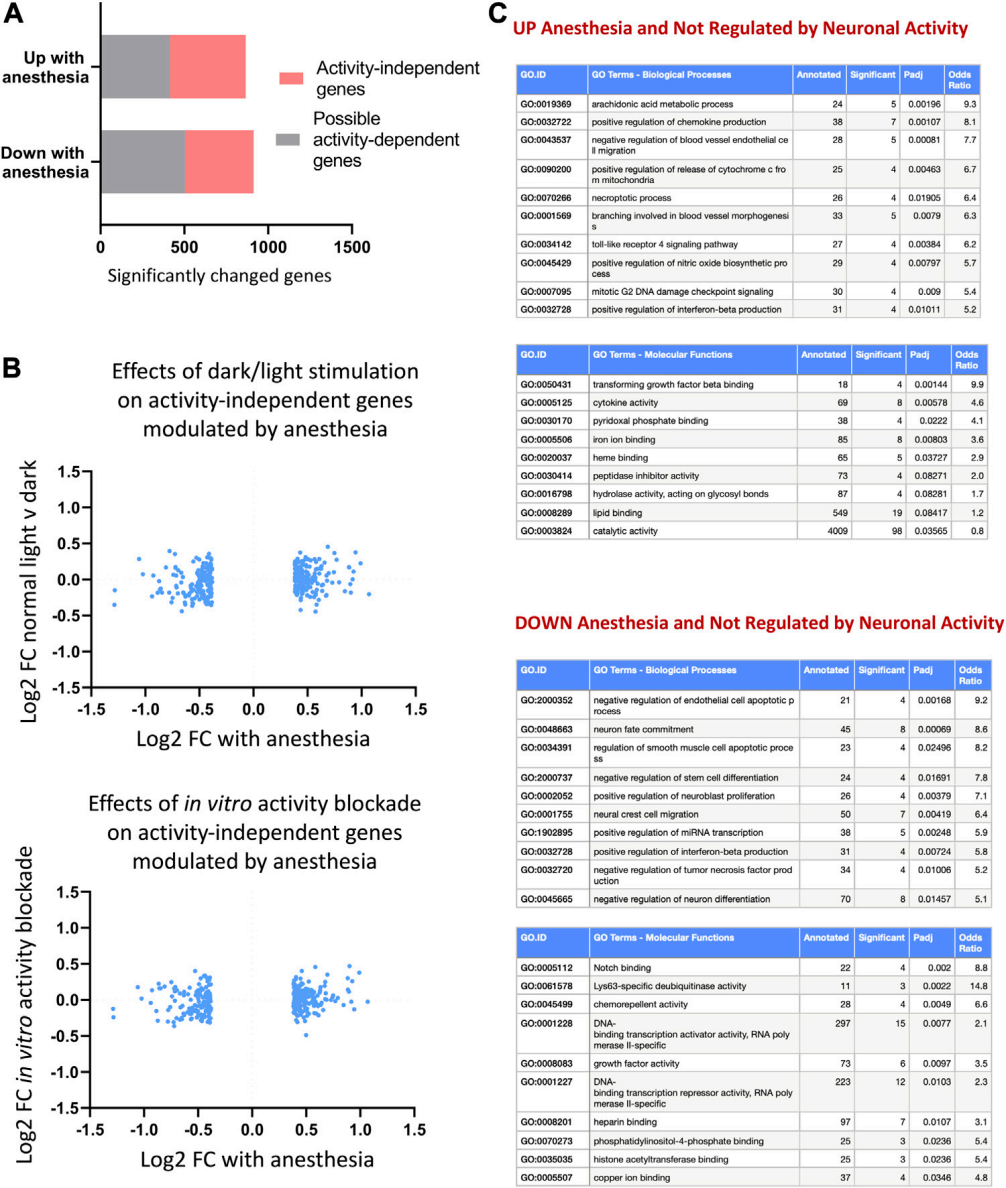
Anesthesia alters genes not regulated by neuronal activity. **(A)** Anesthesia-regulated genes (FPKM cut-off > 1, p_adj < 0.05, FC > 1.3) not regulated by neuronal activity identified by removing possible activity-regulated genes. These are genes found to be changed in normal light v dark conditions, 24 h light-re-exposure or with neuronal-activity blockade *in vitro* (expression cut-off > 1 FPKM, unadjusted *p*-value < 0.1). **(B)** Confirmation that identified non-activity-mediated anesthesia-regulated genes are not altered *in vivo* by visual sensory stimulation or *in vitro* by neuronal-activity blockade. Log2 fold change expression in normal light conditions v darkness (top) or in TTX v control (bottom) shown for activity-independent genes identified as shown in **(A)**. **(C)** Ontological analysis of non-activity-regulated genes induced or repressed by anesthesia. Top ten most significantly enriched pathways are shown, with pathways with less than 4 significant genes or not relevant to tissue type omitted.

We next set out to identify genes altered by anesthesia that are not regulated by neuronal firing. We created a combined list of genes altered by visual sensory experience (changed normal light v dark, or light re-exposure v dark) or changed *in vitro* by blockade of neuronal activity using TTX (expression cut-off > 1 FPKM, unadjusted *p*-value < 0.1). By using a higher unadjusted *p-*value, we ensured a higher threshold to capture all genes that were possibly activity-dependent in any of the stimulation paradigms. We determined genes regulated up or down by anesthesia that were not within this possible activity-dependent set (Figure 5A; Supplementary Data S2-5) and confirmed that anesthesia-regulated activity-independent genes were not altered by light-stimulus or TTX blockade (Figure 5B). Ontological analyses of activity-independent genes upregulated by anesthesia found enrichment for inflammation-associated pathways including chemokine production (*Il4ra, Oas1c, Ticam1, Havcr2, Tlr7, Alox8, Trpv4*), arachidonic acid synthesis (*Aloxe3, Cyp1b1, Alox8, Cyp2s1, Dagla*) and apotosis/necroptosis pathways (*Pmaip1, Plaur, Fas, Bmf, Itpk1, Birc3, Fas, Pygl*) (Figure 5C).

6. TRAPseq allows identification of astrocyte-specific gene-expression changes

Neuronal activity, besides influencing transcription in neurons, also alters transcription in nearby non-neuronal cells (Saab et al., 2016; Hasel et al., 2017; Ioannou et al., 2019). In our previous work, we described how neuronal activity alters transcription in astrocytes to regulate genes involved in astrocyte glycolysis and the astrocyte-neuron lactate shuttle (Hasel et al., 2017), a pathway important for CNS energy provision (Bélanger et al., 2011). We therefore wanted to understand the extent to which anesthesia influenced activity-regulated transcriptional pathways in astrocytes. Given that anesthesia has also been found to influence astrocyte signalling and functions, including altering calcium activity and lactate production, we hypothesised that anesthesia would have both activity-dependent and activity-independent effects.

**FIGURE 6:**
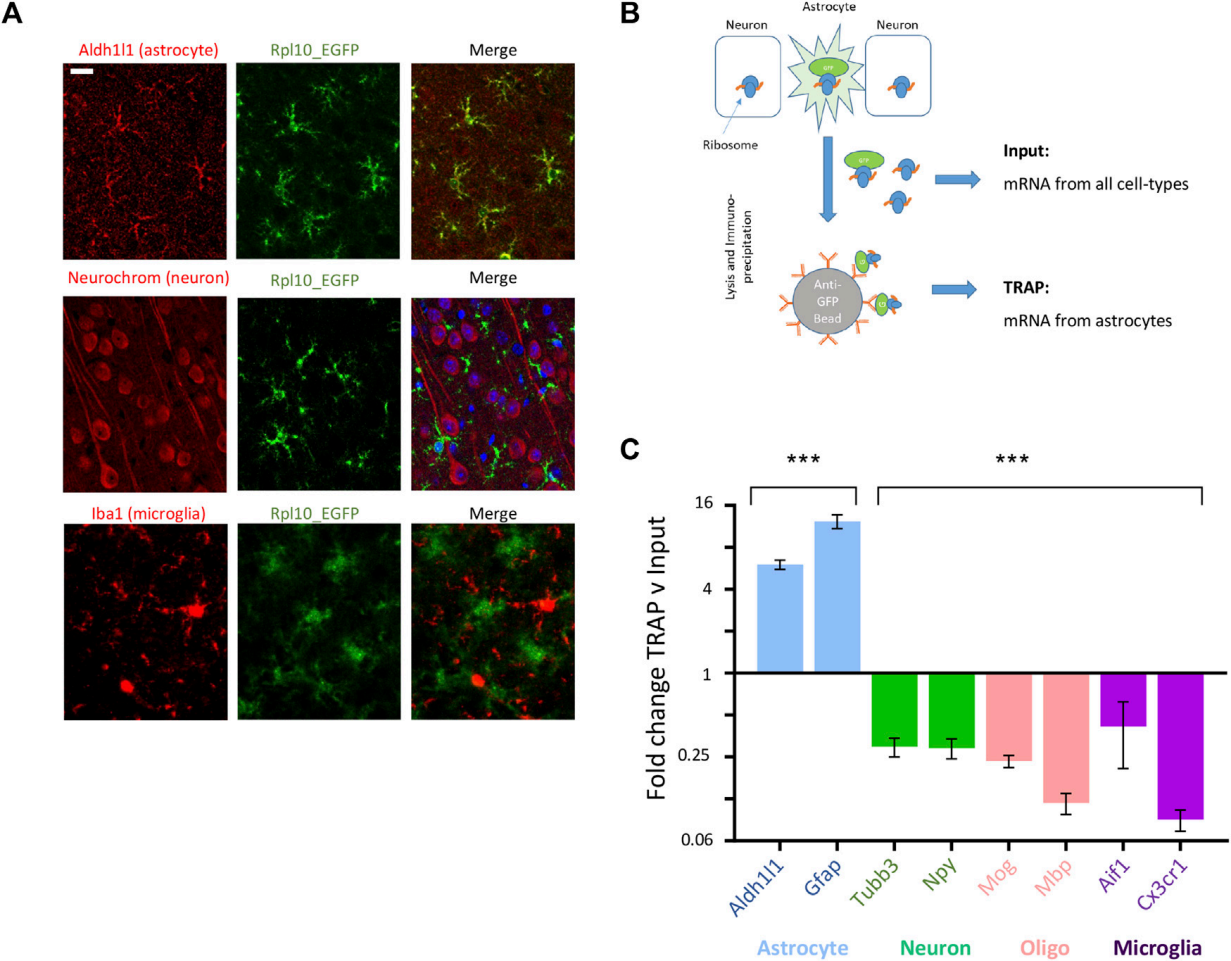
TRAPseq allows determination of astrocyte-specific gene expression. **(A)** Example images from cortical slices from ALDH1l1_eGFP-RPL10a mice with immunofluorescence with anti-EGFP plus an astrocyte-specific marker (Aldh1l1), neuron-specific marker (Neurochrom) and microglial specific marker (Iba1); scale bar 50 uM. **(B)** Schematic of TRAP protocol, allowing isolation of astrocyte-specific mRNA via immunoprecipitation of EGFP-tagged astrocyte ribosomes. **(C)** TRAPseq successfully enriches for astrocyte-specific markers and depletes for marker for other CNS cell-types. Mean fold change enrichment or depletion of cell-type specific gene markers following TRAP v pre-TRAP (input) sample (Mean ± SEM fold change TRAP v input, ***p < 0.001, 1 way ANOVA).

We separated astrocyte-specific gene expression changes from those in other CNS cell-types using translating ribosome affinity purification (TRAP) (Heiman et al., 2014). This uses the transgenic Aldh1l1-EGFP-Rpl13a TRAP transgenic mouse line, where eGFP-tagged ribosomes are expressed specifically in astrocytes through the astrocyte-specific Aldh1l1 promoter. Astrocyte-specific expression of eGFP was confirmed by immunohistochemistry using cell type-specific markers for astrocytes, neurons and microglia (Figure 6A). Tagged ribosomes, along with attached astrocyte-specific translating mRNA, were immunoprecipitated (Figure 6B). Sequencing of post-TRAP mRNA (TRAPseq) confirmed enrichment for astrocyte cell type-specific genes and depletion for markers for other cell types (Figure 6C; Supplementary Figure S11), and hence allowed us to determine astrocyte-specific gene expression changes in our experimental models.

7. Anesthesia alters astrocyte gene expression and upregulates reactivity-associated signatures.

**FIGURE 7:**
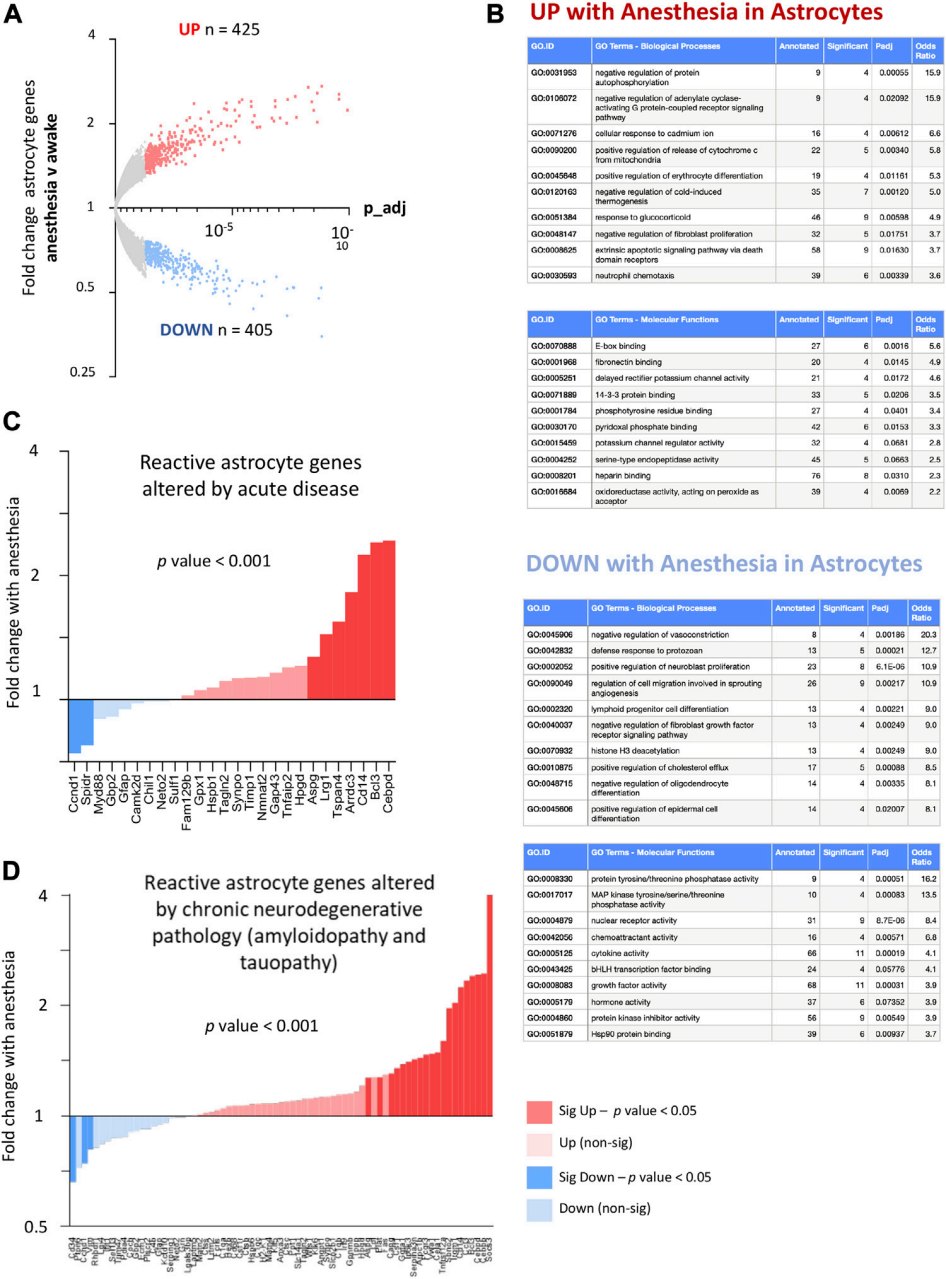
**(A)** Anesthesia alters astrocyte gene expression and upregulates genes associated with both acute and chronic reactive states. Genes significantly induced (red) and suppressed (blue) are highlighted (expression cut-off 1 FPKM, FC > 1.3, p_adj < 0.05) (N = 4 animals per condition). **(B)** Ontological analysis of astrocyte genes induced and repressed by anesthesia. Top ten most significantly enriched pathways are shown, with pathways with less than 4 significant genes or not relevant to tissue type omitted. **(C)** Anesthesia overall upregulates pan-reactive astrocyte genes enhanced by both acute LPS and middle cerebral artery occlusion (MCAO). Gene lists derived from Zamanian et al. (2012), re-derived as described in Jiwaji and Hardingham (2022). Fold change astrocyte genes, anesthesia v control (expression cut-off > 1 FPKM, ratio paired 2-tailed *t*-test). **(D)** Anesthesia overall upregulates reactive astrocyte genes found to be commonly upregulated in end-stage amyloidopathy (APP/PS1 model) and end-stage tauopathy (MAPT-P301S model). Gene-list from Jiwaji and Hardingham (2022). Fold change astrocyte genes, anesthesia v control (expression cut-off > 1 FPKM, ratio paired 2-tailed *t*-test).

To identify how anesthesia altered astrocyte genes, we exposed Aldh1l1-EGFP-rpl13a transgenic mice to 6 h isoflurane and used TRAPseq to isolate astrocyte-specific mRNA. We found that anesthesia altered a wide-programme of gene expression in astrocytes (Figure 7A; Supplementary Data S2-6). Gene ontology analysis of astrocyte genes altered by anesthesia reveals upregulation of genes in stress-response and pro-apoptotic pathways, and downregulation of genes in pathways related to vasoconstriction and cholesterol efflux (*Apln, Dusp5, Cx3cl1, Arhgap42, Nr1h3, Sirt1, Ptch1, Cav1, Pltp*) (Figure 7B).

Astrocytes are known to alter their transcriptional profiles in response to both acute and chronic disease, a term called reactive astrogliosis. Whilst reactive astrogliosis can be defined in several different ways (Escartin et al., 2021), recent work has determined that the transcriptional response of reactive astrocytes can differ depending on the nature and timing of the insult or disease. We wanted to understand to what extent anesthesia altered transcriptional programs in astrocytes which overlap with deleterious astrocyte changes observed during acute and chronic neurodegenerative disease.

First, we compared how anesthesia altered astrocyte genes associated with acute CNS insults. We used published data to identify astrocyte genes upregulated commonly by two different acute insults (acute LPS-mediated inflammation and ischaemia from middle cerebral artery occlusion (MCAO) from data from Zamanian et al. (2012) as described in Jiwaji et al. (2022). We found that anesthesia significantly upregulated the common set of reactive genes increased by both acute LPS administration and MCAO (Figure 7C). Next, we examined how anesthesia alters genes changed during chronic neurodegenerative disease. We identified genes upregulated by two different proteinopathies associated with Alzheimer’s disease (amyloidopathy and tauopathy, as published in Jiwaji et al. (2022)) and found that anesthesia significantly upregulated the common set of astrocyte genes increased by both end-stage amyloidopathy and tauopathy (Figure 7D).

8. Visual sensory-deprivation and light stimulus alter astrocyte gene expression.

**FIGURE 8:**
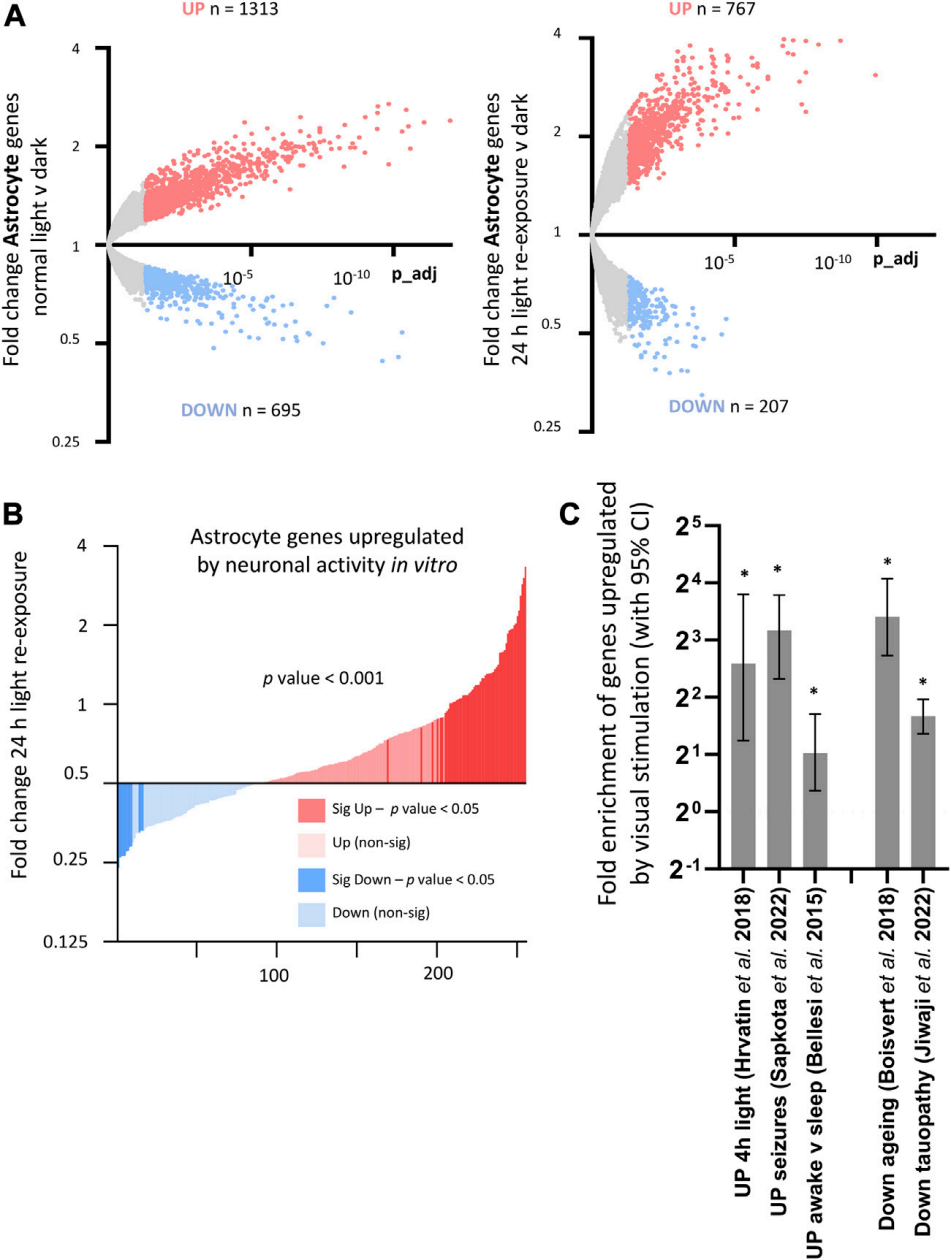
Consequences of visual sensory deprivation and light stimulation on astrocyte gene expression, and comparison with astrocyte genes changed by alternative stimulation paradigms and by ageing and disease. **(A)** TRAPseq identifies astrocyte genes altered by altered visual sensory experience. Genes significantly induced (red) and repressed (blue) are highlighted (expression cut-off > 1 FPKM, FC > 1.3, p_adj < 0.05) for normal light conditions v darkness (left) and with 24 h light-exposure (right). **(B)** Astrocyte genes upregulated by light re exposure overlap with astrocyte genes significantly enhanced by neuronal activity *in vitro* (gene-set from Hasel et al., 2017). Fold change light re-exposure v continuous darkness (expression cut-off > 1 FPKM, ratio paired 2-tailed *t*-test). **(C)** (left). Activity-dependent astrocyte genes upregulated by visual sensory stimulation (Normal light conditions v continuous darkness, expression cut-off > 1 FPKM, FC > 2, p_adj < 0.05) are significantly enriched in sets of astrocyte genes upregulated after 4 h light exposure in single-cell analysis of visual cortex; drug-induced seizures; and in mice experiencing sleep-deprivation v sleep. (*p-value < 0.05, Fisher’s exact test). **(C)** (right). Activity-dependent astrocyte genes are enriched in sets of astrocyte genes reduced by ageing and in a mouse model of tauopathy. (*p-value < 0.05, Fisher’s exact test).

By combining the visual sensory deprivation paradigm (described above) with TRAPseq, we identified astrocyte-specific visual cortex gene expression changes to darkness and light re-exposure. We found that visual stimulation altered the expression of hundreds of astrocyte genes (Figure 8A; Supplementary Data S2-7, 2-8), including upregulating astrocyte genes previously described to be altered by increased neuronal activity *in vitro* (Hasel et al., 2017); (Figure 8B).

Comparing with other published datasets, we found enrichment of astrocyte genes upregulated by visual sensory experience within gene-sets identified by single-cell sequencing analysis of visual cortex genes to 4 h light exposure (Hrvatin et al., 2018), as well as astrocyte genes upregulated in mice experiencing sleep deprivation (Bellesi et al., 2015) and in mice following seizures induced by PTZ (Sapkota et al., 2022) (Figure 8C, left). This was suggestive of a common set of activity-regulated astrocyte genes altered consistently by different stimulus and experimental paradigms. Astrocyte genes increased by sensory stimulus were also significantly enriched within astrocyte gene-sets found to be decreased by ageing (Boisvert et al., 2018) and by neurodegeneration (Jiwaji et al., 2022) (Figure 8C, right), highlighting a potential age/disease related modulation of activity-dependent astrocyte pathways.

9. Anesthesia alters the expression of astrocyte genes regulated by neuronal activity.

**FIGURE 9:**
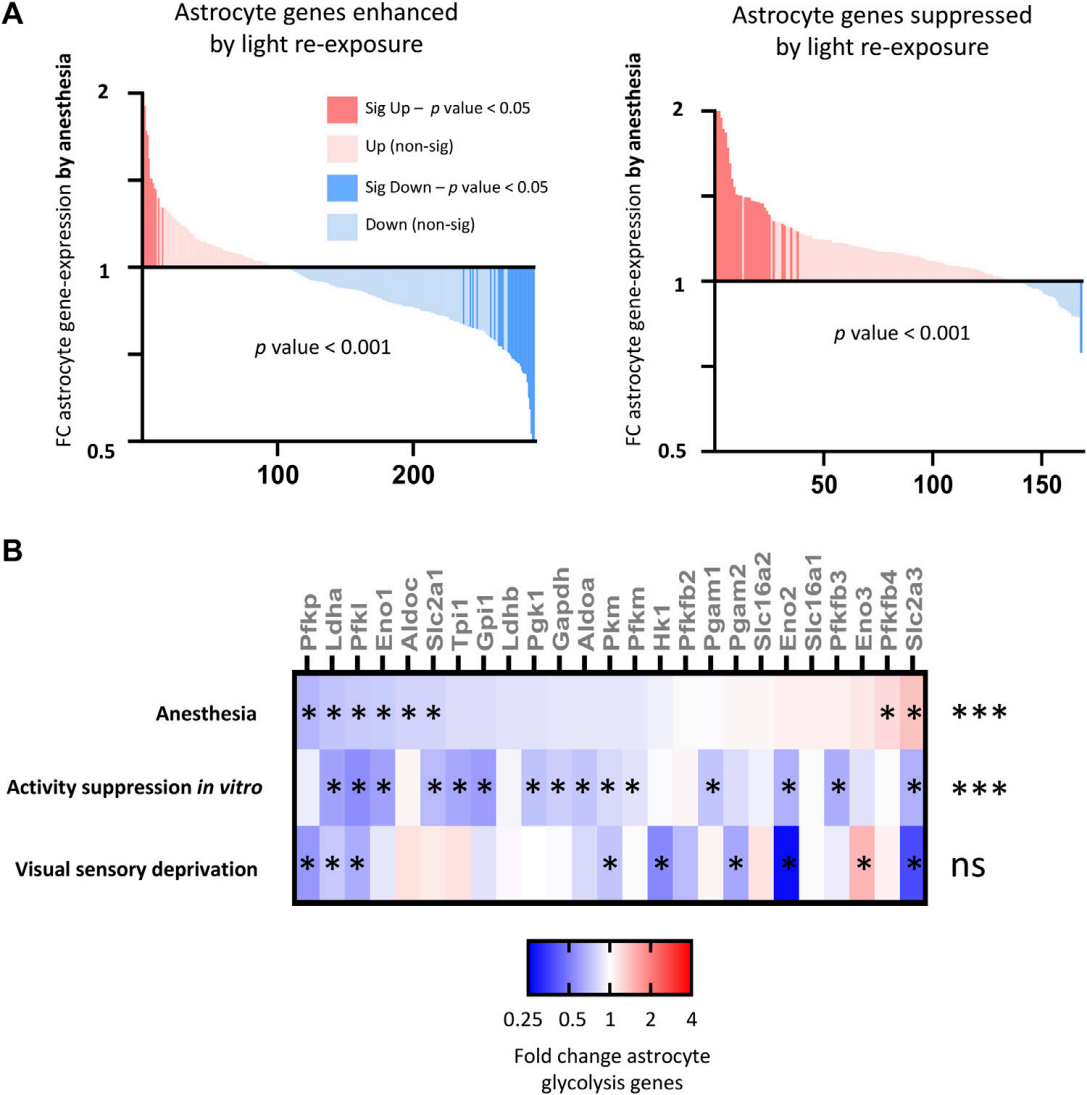
Consequences of anesthesia on astrocyte genes and pathways altered by neuronal activity **(A)** Anesthesia overall downregulates (left) astrocyte genes enhanced by light re-exposure v darkness (expression cut-off > 1 FPKM, FC > 1.5, p_adj < 0.05) and (right) upregulates astrocyte genes suppressed by light re-exposure. Fold change anesthesia v control (expression cut-off > 1 FPKM, ratio paired two-tailed *t-*test). **(B)** Anesthesia downregulates activity-regulated glycolytic genes. Fold change expression of astrocyte glycolytic genes shown for anesthesia v control; *in vitro* expression following suppression neuronal activity with TTX [gene-list from Hasel et al. (2017)]; and visual cortex expression following 6 days of darkness v normal light conditions (expression cut-off > 1 FPKM, *p_adj < 0.05 for each individual gene, ***p-value < 0.001 for overall downregulation of glycolysis gene-set, 2-way ANOVA).

We next investigated how anesthesia altered astrocyte genes identified to be regulated by visual sensory deprivation and stimulation. We found that anesthesia overall significantly downregulates astrocyte genes enhanced by light stimulation, and upregulates astrocyte genes suppressed by light stimulation (Figure 9A).

A key function of astrocytes is to uptake glucose and convert this into lactate via glycolysis. According to the astrocyte neuron lactate shuttle (ANLS) hypothesis this acts as a fuel source for nearby neurons. In prior work, we determined that neuronal activity upregulated the expression of astrocyte glycolysis pathway genes, increasing the ability of astrocytes to uptake glucose and produce lactate. We examined the consequences of anesthesia on astrocyte glycolytic gene expression and found that anesthesia overall suppressed astrocyte glycolytic gene expression down-regulating key glycolytic genes including those for glucose uptake (*Slc2a1*), rate-limiting genes for glycolysis (*Pfkp, Pfkl, Pfkm*), and genes regulating lactate production (*Ldha, Ldhb*) (Figure 9B). However, activity-suppression through dark-exposure did not have a significant consequence on astrocyte glycolytic gene expression.

10. Anesthesia alters non-activity dependent astrocyte genes, upregulating astrocyte pathways associated with inflammation and oxidative stress.

**FIGURE 10:**
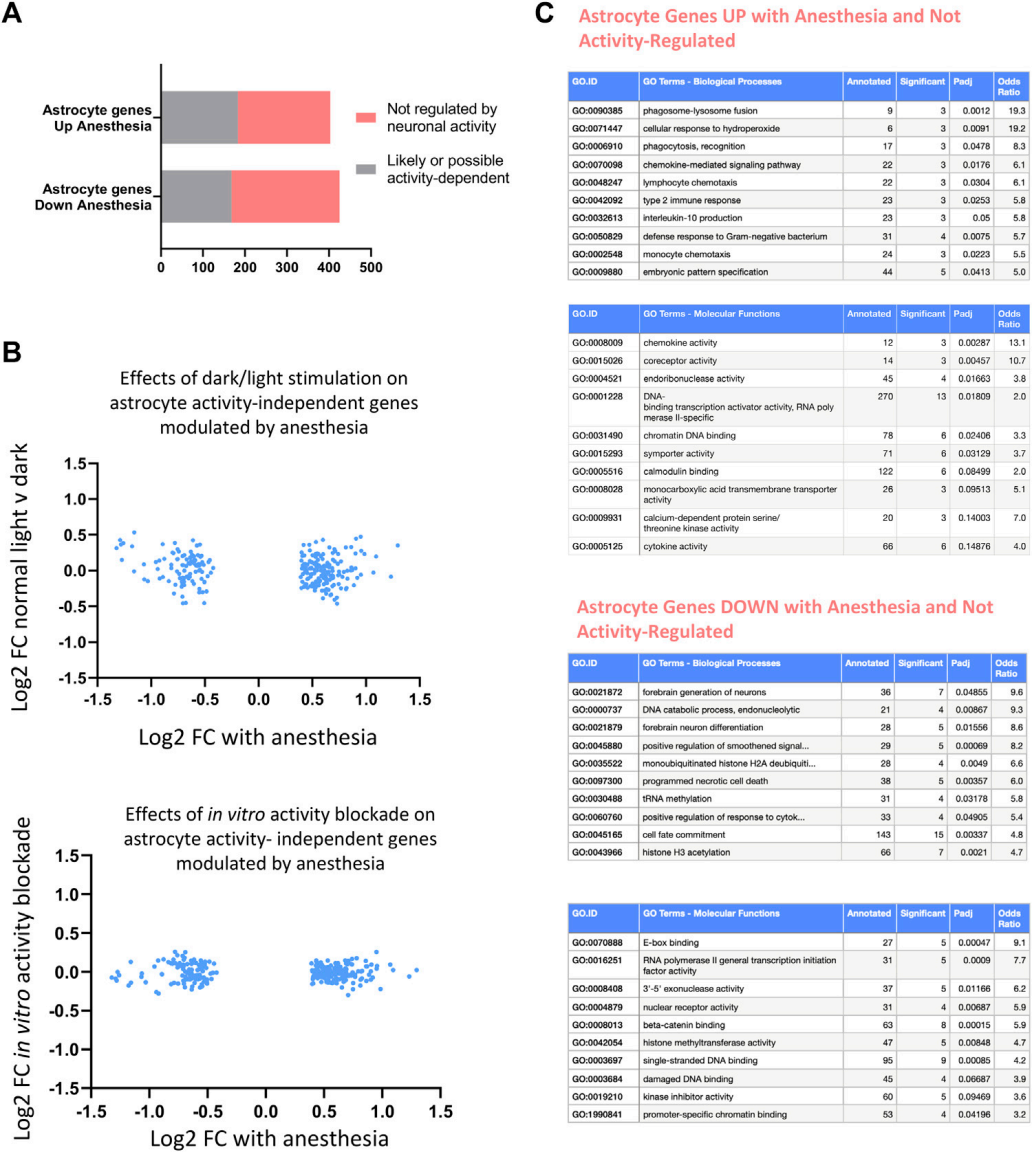
Consequences of anesthesia on astrocyte genes not regulated by neuronal activity. **(A)** Astrocyte anesthesia-regulated genes (expression cut-off > 1 FPKM, p_adj < 0.05, FC > 1.3) not regulated by activity identified by removing possible activity-regulated genes as those changed in normal light v dark conditions, 24 h light-re-exposure or with neuronal-activity blockade *in vitro* (expression cut-off > 1 FPKM, unadjusted *p*-value < 0.1). **(B)** Identified astrocyte non activity-mediated anesthesia-regulated genes are not altered *in vivo* by visual sensory stimulation or *in vitro* by neuronal-activity blockade. Log2 FC expression of astrocyte genes normal light conditions v darkness (top) or in TTX v control (bottom) shown of genes identified in **(A)**. **(C)** Ontological analysis of astrocyte non-activity-regulated genes induced or repressed by anesthesia. Top ten pathways are shown, with pathways with less than 3 significant genes or fold enrichment < 2 omitted.

Finally, we determined to what extent anesthesia altered astrocyte gene expression independent of activity-mediated effects. Using an identical approach to that described earlier in the paper, we created a combined list of genes altered (expression cut-off > 1 FPKM, unadjusted *p*-value < 0.1) in astrocytes by visual sensory experience (normal light v dark, or with light re-exposure) or *in vitro* by manipulation of neuronal activity using TTX [data from Hasel et al. (2017)], using a higher unadjusted *p*-value to capture any genes that were at all likely to be activity-dependent in all paradigms (Figure 10A; Supplementary Data S2-9). We confirmed that activity-independent astrocyte genes altered by anesthesia did not change both in response to visual sensory-deprivation or following *in vitro* alteration of neuronal activity (Figure 10B). Ontology analysis of activity-independent astrocyte genes altered by anesthesia (Figure 10C) revealed predominantly an upregulation of inflammation-associated pathways, including those regulating cytokine production (*Lefty1, Ccl17, Ccl3, Wnt10a, Ccl12, Wnt4*), and upregulation of pathways for phagosome-lysosome fusion and immune responses.

## Discussion

### Anesthesia alters a wide programme of cortical transcription

Although general anesthesia is widely used in humans and animals, the molecular consequences remain poorly understood. Developing a better understanding of the biological pathways altered by anesthesia is especially important because of negative associations in both pre-clinical and clinical contexts. In animals, anesthesia increases brain cell apoptosis (Yon et al., 2005), alters mitochondrial properties (Zhang and Xie, 2012), and worsens neuropathology in models of Alzheimer’s disease (Perucho et al., 2010). In clinical studies, anesthesia has been linked to post-operative delirium and long-lasting cognitive impairment (Bratzke et al., 2018).

In this study, we used RNA-sequencing approaches to map the consequences of prolonged volatile anesthesia on cortical transcription. The sensitivity of our methods revealed hundreds of cortical genes that are altered during anesthesia, significantly expanding on the number of genes previously identified with less sensitive methods such as microarray analysis. Genes upregulated by anesthesia are enriched for ontology terms related to inflammation and apoptosis, supporting a detrimental role for prolonged anesthesia on cortical function.

### Anesthesia alters astrocyte transcription

The primary target of anesthetic agents has been presumed to be neurons, where they are widely considered to inhibit synaptic firing through GABA receptor activation or NMDA receptor inhibition. However, in our study, we determined that anesthesia has consequences beyond only neurons. We identified transcriptional consequences on total cortical gene expression, as well as focusing on gene expression changes occurring specifically in astrocytes. Astrocytes, the most common non-neuronal cell type in the CNS, are important for several neurosupportive roles including maintaining neurotransmitter, ionic, metabolic and antioxidant homeostasis. Anesthesia has previously been described to act on astrocytes via astrocyte GABA receptors (Chung et al., 2021), and altering astrocyte calcium signalling (Thrane et al., 2012) and gap junction properties (Liu et al., 2016). However, the transcriptional consequences of these effects on astrocyte pathways important for brain function were unknown. In this study, using TRAP-sequencing to isolate astrocyte-specific gene expression changes from those in other CNS cell types, we present a detailed description of how anesthesia alters astrocyte transcription, further expanding our understanding of the molecular consequences of anesthesia on non-neuronal CNS cell types.

We found that the transcriptional changes induced by anesthesia in astrocytes overlap with reactive changes seen in response to both acute insults (LPS administration and acute ischaemia through MCA occlusion) and chronic insults (neurodegenerative amyloid and tau pathology). Indeed, the fact that 6 h anesthesia induced reactive changes in astrocytes previously identified in response to chronic dementia-associated pathology highlights the potential deleterious consequences of anesthesia, especially in those with pre-existing neurodegenerative disease.

### CNS and astrocyte activity-responsive genes identified by visual sensory deprivation and light stimulation

It is widely believed that the primary mechanism of anesthesia function is through global suppression of neuronal activity through GABA activation or NMDA-receptor inhibition. Altered neuronal activity has been described by ourselves and others to alter transcriptional networks in both neurons and astrocytes to modulate pathways important for neuronal and brain homeostasis and health. Therefore, the consequence of general anesthesia on these activity-dependent transcriptional pathways was of particular interest to us.

In this study, using visual sensory deprivation and light stimulation paradigms, we have revealed how altered neuronal activity regulates CNS transcription in non-anesthetized animals. We have identified hundreds of genes (across all cell-types and specifically in astrocytes) that are regulated by visual sensory stimulation. The sensitivity and added transcriptional depth possible through the TRAP-seq approaches used adds to the smaller number of astrocyte and neuronal genes previously identified to be altered by light stimulation using single-cell approaches (Hrvatin et al., 2018). Furthermore, we found that visually responsive astrocyte genes overlap with astrocyte genes altered in other studies that involved dysregulated neuronal activity such as seizures (Sapkota et al., 2022) and sleep-deprivation (Bellesi et al., 2017), consistent with a common set of activity-responsive astrocyte genes. We also found that astrocyte genes upregulated by activity are enriched in sets of astrocyte genes found to be reduced by ageing (Boisvert et al., 2018) and neurodegeneration (Jiwaji et al., 2022). As activity-dependent astrocyte pathways are essential for metabolic homeostasis, it would be interesting to understand the extent to which reductions in these pathways contribute towards brain metabolic dysfunction observed in both ageing and degenerating brains.

### Anesthesia dysregulates activity-dependent genes, suppressing activity-regulated homeostatic pathways and enhancing activity-suppressed pro-death pathways

In keeping with the proposed mechanism of action as an activator of GABA and inhibitor of NMDA receptors, we determined that anesthesia is a strong suppressor of activity-regulated CNS and astrocyte genes. This includes suppression of activity-regulated neuroprotective and pro-synaptogenesis genes (*Bdnf*, *Arc, Npas4*, *Homer1*). Indeed, the negative consequences of anesthesia on suppressing activity-dependent genes extends beyond the level of activity-suppression possible by visual-sensory deprivation and is only revealed by examining genes found to be regulated in conditions of total neuronal activity blockade using TTX *in vitro*. Comparing genes that are changed by total activity blockade revealed that anesthesia upregulates activity-suppressed pro-death genes and suppresses activity-enhanced genes in astrocytes responsible for CNS metabolic homeostasis. The fact that these detrimental effects are not seen in visual-sensory deprivation is of course not unsurprising, given that eye-closure or sleeping is not associated with the harmful effects attributed to anesthesia.

Overall, these findings reveal how anesthesia suppresses activity-dependent pathways in the cortex, leading to reduced expression of genes associated with brain homeostasis and boosting genes associated with cell-death. These changes may contribute to observed deleterious consequences of anesthesia, especially as dysregulation of brain metabolism has been demonstrated to occur in patients with delirium using nuclear imaging (Haggstrom et al., 2017). Our follow-on work now seeks to understand the consequences of targeting restoration of activity-dependent pathways during anesthesia to reduce deleterious transcriptional changes for therapeutic benefit.

### Activity-independent consequences of anesthesia drive deleterious and inflammation-associated responses

Beyond consequences on activity-dependent gene expression, we also identified that anesthesia altered expression of hundreds of CNS and astrocyte genes that were not modulated by neuronal firing as identified by visual sensory deprivation or *in vitro* blockade of neuronal activity. These findings are consistent with anesthesia having wider consequences on CNS beyond simply one of acting as a pharmacological inhibitor of neuronal activity.

We identified activity-independent consequences of anesthesia on pathways related to apoptosis and inflammation responses, including upregulating pathways related to mitochondrial-associated apoptosis pathways and lipoxygenase production. In astrocytes, we found that anesthesia alters the expression of non-activity regulated genes related to inflammation including those regulating phagosome-lysosome function, chemokine signalling and cytokine production. These findings are consistent with anesthesia driving further deleterious and inflammation-associated responses in neuronal and non-neuronal cells beyond those detrimental effects seen by neuronal activity suppression alone.

The extent of inflammation-associated changes driven by anesthesia that are independent of activity-regulated effects points towards a broader pro-inflammatory role of anesthesia on CNS cells. Indeed, anesthesia has been postulated to have immune modulating effects, and the mechanisms that drive these deleterious pro-inflammatory effects (including upstream transcription factors and regulators) now requires to be studied. Furthermore, microglia-astrocyte interactions have been shown to be important in regulating astrocyte responses to inflammation (Liddelow et al., 2017). Therefore, it is likely that non-activity mediated changes of anesthesia on astrocytes may be downstream of uncharacterised microglia responses. A fuller understanding of the interactions between astrocytes and microglia (as well between astrocytes and other non-cellular elements within the brain milieu) during anesthesia may reveal important pathways that could be targeted to reduce the potentially deleterious inflammatory consequences of anesthesia identified in this work.

## Limitations of work

An important limitation is that the data presented in this work represents changes at the level of gene expression, which may not translate to protein-level or functional consequences. The use of the translating ribosome affinity purification methodology mitigates this somewhat, as this will only sequence mRNA molecules that are being actively translated. Hence, these are more likely to represent gene expression changes reflecting changes to protein synthesis. Furthermore, we have previously determined that alterations in activity-controlled apoptosis genes by suppression of neuronal activity did lead to changes in protein concentrations (Léveillé et al., 2010). However, our work will not identify changes to cellular functions by anesthesia through controls downstream of protein synthesis (for example, changes in protein degradation pathways). Our work has investigated the consequences of a single anesthesia agent (isoflurane) on CNS and astrocyte pathways. The extent to which consequences of using other anesthesia agents overlap with our findings remains to be determined. Finally, our work does not address the long-term consequences of anesthesia on brain and astrocyte gene-expression. Ongoing work now aims to investigate how gene-expression perturbations persist following recovery from anesthesia, and the extent to which these may drive long-lasting impairments in cognition.

## Concluding remarks

Our study provides a comprehensive insight into the transcriptional consequences of anesthesia on global CNS and astrocyte transcription. By identifying how anesthesia alters genes that are both activity-dependent and activity-independent, we have identified stress and inflammation-associated responses associated with loss of metabolic homeostasis and enhancement of pro-death pathways. This rich dataset will provide a valuable resource for researchers using anesthesia in brain research as well as those exploring mechanisms behind the clinically-associated cognitive complications of anesthesia.

## Data Availability

The datasets presented in this study can be found in online repositories. The names of the repository/repositories and accession number(s) can be found below: https://www.ebi.ac.uk/biostudies/arrayexpress/studies/E-MTAB-13054.

## Supplementary material

SUPPLEMENTARY FIGURE S3 Consequences of anesthesia on genes suppressed by visual sensory stimulation. Genes suppressed by normal light conditions **(A)** or light re-exposure **(B)** compared to darkness (expression cut-off > 1 FPKM, padj < 0.05, FC > 2, ratio paired 2-tailed *t*-test) are not overall down-regulated or upregulated by anesthesia.

SUPPLEMENTARY FIGURE S11 qtPCR confirmation of selected genes determined to be altered by RNAseq analysis throughout manuscript.**(A)** qtPCR validation of anesthesia-mediated suppression of *Arc* and *Bdnf*. (Mean ± SEM fold change RNA expression v 6 h anesthesia normalised to house-keeping gene *Rpl13a*, N = 5 animals per condition, **p* value < 0.05, two-tailed *t*-test). **(B)** qtPCR validation of visual sensory deprivation-mediated suppression of *Arc* and *Bdnf*. (Mean ± SEM fold change RNA expression v 7 days darkness normalised to house-keeping gene *Rpl13a*, N = 4 animals per condition, **p* value < 0.05, 1-way ANOVA with multiple comparisons). **(C)** qtPCR validation of TRAP-mediated enrichment or depletion of cell type-specific markers (Mean ± SEM fold change RNA expression v input sample normalised to house-keeping gene *Rpl13a*, N = 3 animals per condition, **p* value < 0.05, 1-way ANOVA for enriched or depleted gene-sets).
