# Supplementary figures and images for: General anesthesia alters CNS and astrocyte expression of activity-dependent and activity-independent genes
Source: Front Netw Physiol. 2023 Aug 21;3:1216366. doi: 10.3389/fnetp.2023.1216366 (PMC10476527; doi:10.3389/fnetp.2023.1216366)

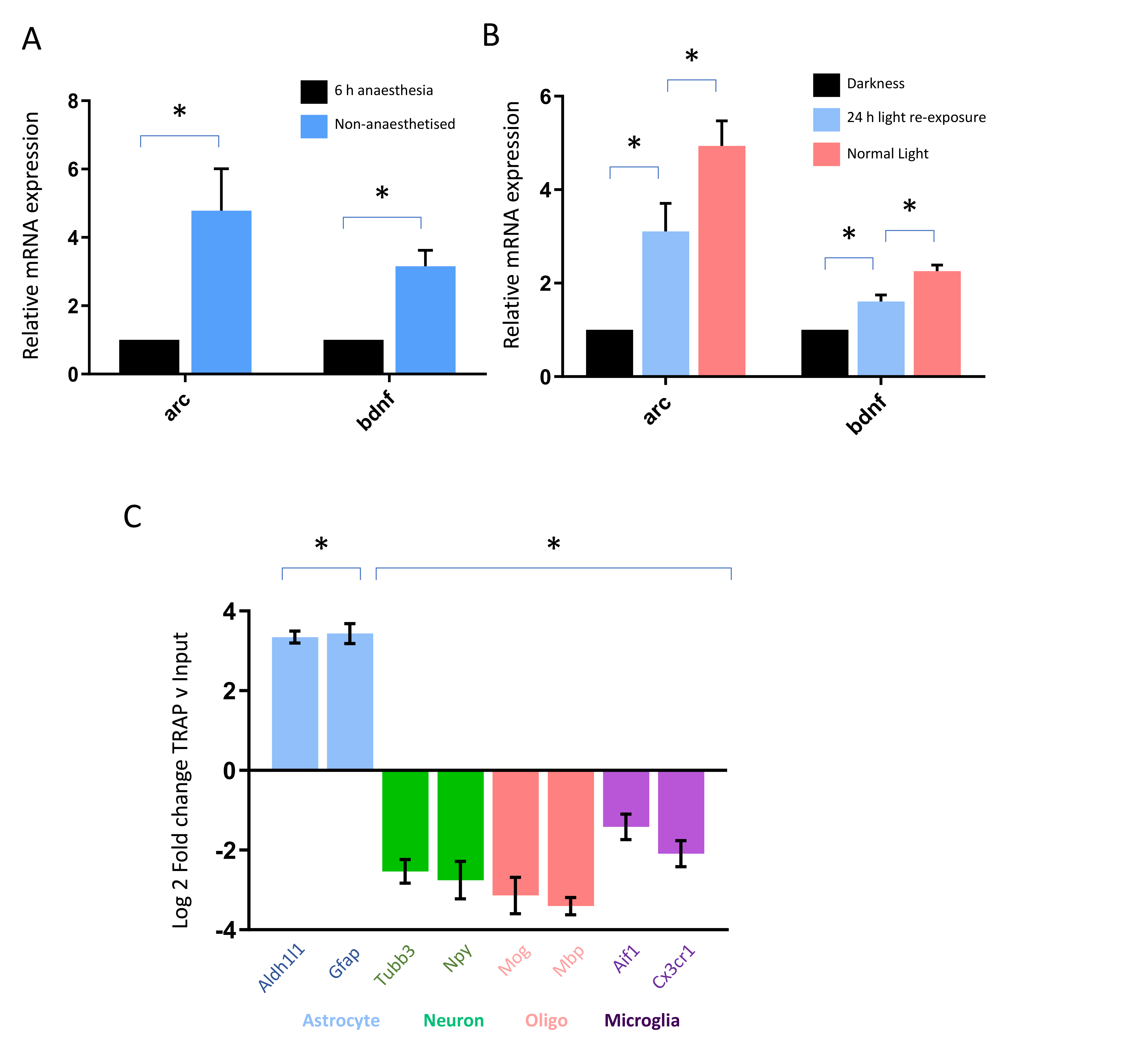

Supplement: Supplementary file 2 [file Image2.tif]

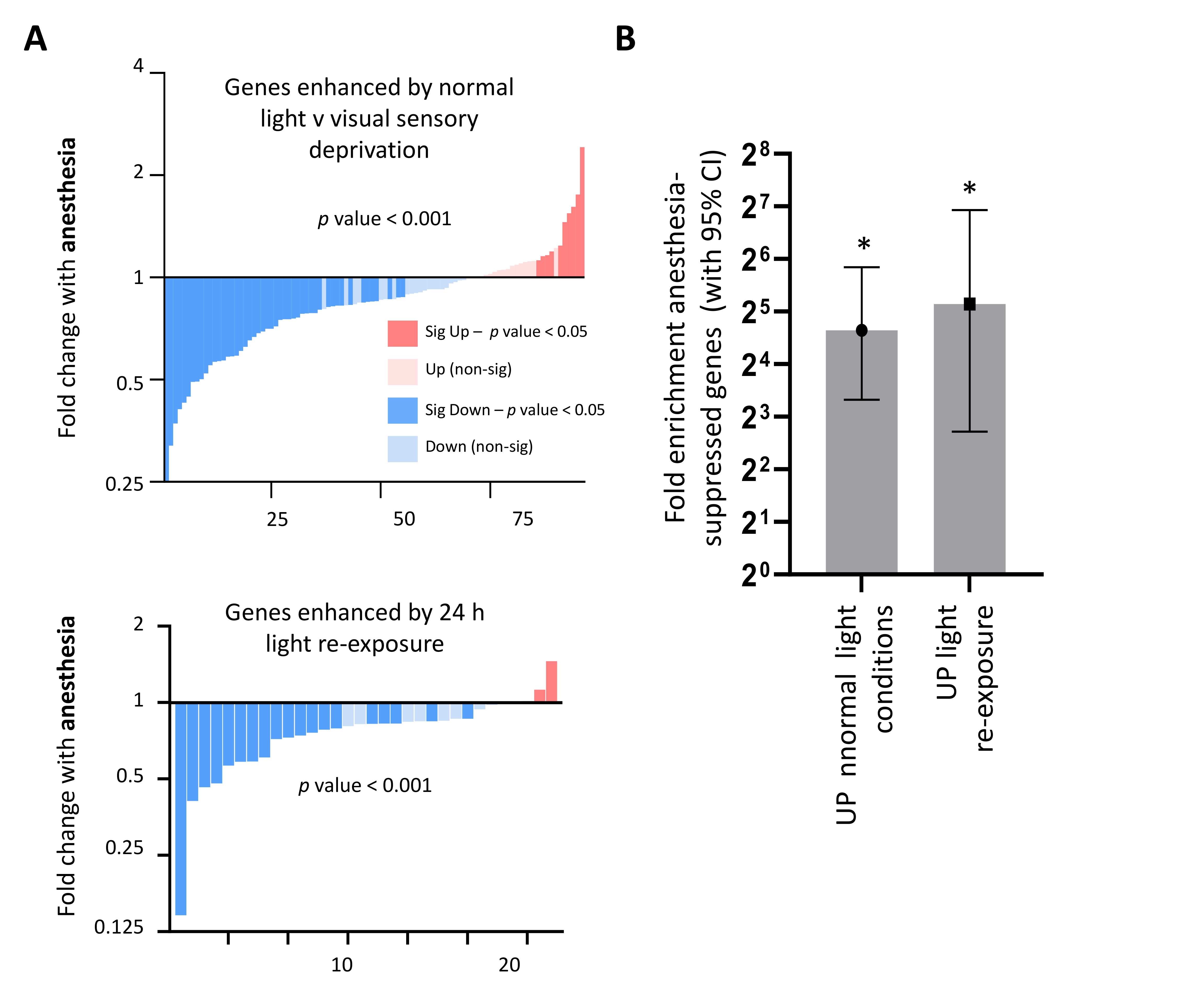

Supplement: Supplementary file 3 [file Image1.tif]
